# ‘The tabloid test’: a qualitative interview study on the function and purpose of termination of pregnancy review committees in Victoria, Australia

**DOI:** 10.1186/s12978-023-01624-w

**Published:** 2023-07-18

**Authors:** Hilary Bowman-Smart, Louise Keogh, Casey M. Haining, Anne O’Rourke, Lachlan de Crespigny, Julian Savulescu

**Affiliations:** 1 Murdoch Children’s Research Institute, 50 Flemington Rd, Parkville, VIC 3052 Australia; 2 Monash Bioethics Centre, Monash University, Clayton, VIC Australia; 3 Ethox Centre, University of Oxford, Oxford, UK; 4 Centre for Health Equity, Melbourne School of Population and Global Health, University of Melbourne, Parkville, VIC Australia; 5 Monash Business School, Monash University, Clayton, VIC Australia; 6 Uehiro Centre for Practical Ethics, University of Oxford, Oxford, UK; 7 Centre for Biomedical Ethics, Yong Loo Lin School of Medicine, National University of Singapore, Singapore, Singapore; 8 Australian Centre for Precision Health, University of South Australia, Adelaide, Australia; 9 Medicine, Dentistry and Health Sciences, University of Melbourne, Parkville, VIC 3052 Australia

**Keywords:** Reproductive healthcare, Termination of pregnancy, Abortion, Qualitative research, Australia

## Abstract

**Background:**

Termination of pregnancy (TOP) is not an uncommon procedure. Availability varies greatly between jurisdictions; however, additional institutional processes beyond legislation can also impact care and service delivery. This study serves to examine the role institutional processes can play in the delivery of TOP services, in a jurisdiction where TOP is lawful at all gestations (Victoria, Australia). As per the *Abortion Law Reform Act 2008*, TOPs post-24 weeks require the approval of two medical practitioners. However, in Victoria, hospitals that offer post-24 week TOPs generally require these cases to additionally go before a termination review committee for assessment prior to the service being provided. These committees are not stipulated in legislation. Information about these committees and how they operate is scarce and there is minimal information available to the public.

**Methods:**

To trace the history, function, and decision-making processes of these committees, we conducted a qualitative interview study. We interviewed 27 healthcare professionals involved with these committees. We used purposive sampling to gain perspectives from a range of professions across 10 hospitals. Interviews were transcribed verbatim, identifying details removed and inductive thematic analysis was performed.

**Results:**

Here, we report the three main functions of the committees as described by participants. The functions were to protect: (1) outward appearances; (2) inward functionality; and/or, (3) service users. Function (1) could mean protecting the hospital’s reputation, with the “Herald Sun test”—whether the TOP would be acceptable to readers of the Herald Sun, a tabloid newspaper—used as a heuristic. Function (2) related to logistics within the hospital and protecting the psychological wellbeing and personal reputation of healthcare professionals. The final function (3) related to ensuring patients received a high standard of care.

**Conclusions:**

The primary functions of these committees appear to be about protecting hospitals and clinicians within a context where these procedures are controversial and stigmatized. The results of this study provide further clarity on the processes involved in the provision of TOPs at later gestations from the perspectives of the healthcare professionals involved. Institutional processes beyond those required by legislation are put in place by hospitals. These findings highlight the additional challenges faced by patients and their providers when seeking TOP at later gestations.

## Plain language summary

Abortion can be difficult to access. In Victoria, Australia, under the law, abortion is allowed at any time during a pregnancy—although after you have been pregnant for more than 24 weeks, the approval of two doctors is required. However, hospitals in Victoria that offer late abortions require more than the approval of two doctors. Hospitals have put in place committees that review each case and make a decision about whether the hospital will provide the abortion. There is not a lot of information about these committees—we do not know exactly why they exist, what they are for, or how they work. To find out, we interviewed doctors and other healthcare professionals (like midwives) who were involved in these committees. In this paper, we report the reasons these people gave for why the committees exist and what they are for. There were three main reasons. The first purpose of the committee is so the hospital does not get criticised in newspapers or by other people outside the hospital for performing these late abortions. The second reason is to help and protect those inside the hospital. For example, having a committee means that the doctors do not have to make the decisions themselves. People also said that the committees think about how the staff are feeling. The third reason is so that the hospitals provide the best care they can, and that they can continue to provide late abortions in the future. With this study, we found out some more important information about these committees that we did not have before. What we found shows that it is not just the law that matters—other things can also affect whether you can get an abortion.

## Introduction

Termination of pregnancy (TOP), or abortion, is not an uncommon procedure. However, it is subject to a significant amount of stigma (which tends to increase with gestation), with many jurisdictions restricting access to TOP beyond particular gestations, or outright banning it [1, 2]. Regulation of TOP as a procedure is currently undergoing significant changes across the world. The 2022 overturn of *Roe v Wade* in the United States has led to several states, such as Texas, enacting legislation significantly restricting TOP access; in other states such as New Mexico, which borders Texas, TOP is legal at all gestations [3, 4]. Countries such as Poland and El Salvador have enacted what amount to de facto bans on TOP [5]. Legislation has also been used to restrict access to TOP in other ways, such as through “TRAP” (Targeted Regulation of Abortion Providers) laws which impose costly (and medically unnecessary) requirements for facilities providing abortions [6]. There are also non-legislative barriers to TOP access, which can include financial, cultural, and geographic barriers [7]. Institutional factors such as interpersonal workplace dynamics, as well as institutional processes, can also impact service delivery but are less explored in the literature [8]. In this paper, we focus on institutional processes, and the role they can play in the delivery of TOP services. These results have implications for other jurisdictions, and provide insight into the ways provision of TOP services, particularly later in gestation, can be shaped by processes beyond legislation.

TOPs are performed at a range of gestations and for a number of reasons, with the vast majority of TOPs taking place before 12 weeks gestation. However, a very small number of TOPs occur at later gestations (e.g. post-23 or -24 weeks). While there does not appear to be a significant amount of data, generally TOP procedures at later gestations (e.g. post-24 weeks) are estimated to constitute less than 1% of all TOPs [9]. Most frequently, these are done because of the presence of fetal anomaly [10]. Particularly in the case of “wanted” pregnancies, the decision to have a TOP at a later gestation can be a traumatic or distressing experience for patients and their partners [11, 12]. TOP in cases where there is no known fetal anomaly may also be sought for a variety of psychosocial reasons, which frequently include financial and social precarity but can also include incest and rape [13–15]. Requests for TOP for psychosocial reasons at later gestations may further be the result of needing more time to make the decision, changed circumstances, being unaware of the pregnancy, or mistaking the gestation [16].

### The legislative context

In Australia, TOP is governed at the state and territory level rather than at the federal level. As of 2021, TOP has been decriminalised (at least partially) in all Australian jurisdictions; however, the access requirements vary amongst jurisdictions [17]. Furthermore, even post-decriminalisation, a range of barriers can remain, particularly for marginalised populations [18]. Since the enactment of the *Abortion Law Reform Act 2008*, the state of Victoria has no gestational limit on access to TOP [19]. Until 24 weeks’ gestation, TOP is available on request without additional requirements. After 24 weeks, the law requires two medical practitioners to reasonably believe that the TOP is “appropriate in all the circumstances” [19]. In making such a determination, the legislation requires that regard is given to “all relevant medical circumstances” and the “woman’s current and future physical, psychological and social circumstances” [19]. For the purposes of this article, Victorian TOPs post-24 weeks will hereafter be referred to as late terminations of pregnancy (LTOPs).

Despite the Victorian law only requiring the approval of two medical practitioners for LTOPs to be lawfully performed, public hospitals that perform these terminations in Victoria have introduced an intra-hospital form of regulation through the establishment of termination review panels or committees (TRCs) [20]. These committees are not required by law but have been implemented at the institutional level, and can vary between institutions in terms of internal structure and processes. LTOPs in Victoria are generally accessed through the public system; LTOP in the private system has become less accessible [21]. Furthermore, while there is a move towards geographic decentralisation of the provision of medical TOP in the first and second trimester of pregnancy, access is still restricted in rural and regional areas [22–24]. However, more complex cases (such as LTOP) frequently require travel to Melbourne, the capital city of Victoria.

The implementation of TRCs or similar processes is not unique to Victoria. For example, one study reported the use of such committees in the Australian states of Queensland and New South Wales, although the study was conducted prior to decriminalisation in the respective states [25]. Review by a committee or panel has also been described in international contexts, such as France and Israel [26, 27]. Despite the existence of such committees, as is the case in Victoria, Australian abortion legislation largely remains silent with respect to TRCs. The exception is Western Australia, where TOPs after 20 weeks’ gestation need to be approved by at least two medical practitioners from a ministerially appointed panel comprising at least six medical practitioners; these medical practitioners have to agree that the mother (or unborn child) has a severe medical condition that justifies the procedure [28, 29].The current New South Wales legislation also refers to TRCs, with respect to TOPs performed post-22 weeks, whereby clinicians may (but are not required to) seek advice from a multi-disciplinary team or hospital advisory committee [30].

The impetus for the creation of the TRCs at two major tertiary hospitals in Melbourne has been described in the literature as a controversial case involving the provision of an LTOP at the Royal Women’s Hospital in 2000 [20]. A woman attended the Royal Women’s Hospital at 31 weeks’ gestation after her fetus had been diagnosed with a skeletal dysplasia. After this diagnosis, the woman indicated that she would “kill herself or do anything not to have the baby” [31]. LTOP was performed at 32 weeks’ gestation [31]. This case, hereafter referred to as ‘the Case’, came to the attention of a federal politician, the (now former) Senator Julian McGauran, who opposed TOP [32]. Several of the medical practitioners involved were either fired or suspended from their positions. Senator McGauran reported the doctors to the Medical Practitioners Board, and the complaint was not resolved until 2006, when no evidence of unprofessional conduct was found [33]. The LTOP was also referred to the Victorian Coroner, due to a lack of clarity around the legality of providing the LTOP [20, 31]. The Coroner determined that they did not have jurisdiction to investigate, as the baby was stillborn [31]. One of the authors of this paper (LDC) is a medical practitioner who was involved in the Case.

The function of TRCs has previously been described by Woodrow as a means for avoiding liability for the clinician (serving the interests of doctors) and providing public accountability; Woodrow also states that at one hospital, the creation of a TRC was pushed by hospital administration, and at the other, the initiative came from a group of clinicians [20]. However, overall, the literature on TRCs in Victoria is thin, with the one paper by Woodrow being published in 2003 [20]. Australian women have reported access to TOP as inaccessible and confusing, even when they know they have the right to access the procedure [34]. Therefore, it is timely to review how these committees work and the role they play in TOP provision.

Since Woodrow’s examination [20], more hospitals have created TRCs. Furthermore, as the most recent literature is from nearly two decades ago, their purpose and function may have evolved in that time. Therefore, to address this gap, we conducted a qualitative interview study with healthcare professionals involved in TRCs in Victoria. Our study aimed to capture more details about the TRCs, including information about their purpose, decision-making, and general processes. The main focus of this paper is to provide insight into the purpose and function of TRCs.

## Methods

Given the paucity of literature describing the process of LTOP service delivery in Victoria, a qualitative design was chosen. This study adopted a phenomenological methodology as it provided an opportunity for participants to reflect on their lived experiences relating to the TRCs [35]. The population selected for this study were healthcare professionals who had direct experience of a TRC in a Victorian hospital, either through making a referral, counselling patients, presenting cases to the TRC, or being a member of a TRC. These individuals were deemed to be most knowledgeable of the process and best placed to describe the purpose and function of the committees. Semi-structured interviews were chosen because they are useful for capturing previously unaddressed perspectives [36]. They do, however, require some level of background knowledge of the subject to cover likely key topics [37]; this background knowledge was obtained through the researchers’ previous work on TOP in Victoria, and involvement in the provision of LTOP in the clinical setting.

Ethics approval was obtained from Monash University HREC [MUHREC Project 13334].

### Sampling and recruitment

Purposive snowball sampling was performed to ensure that our sample included participants from multiple hospitals (ten hospitals overall), as well as a diversity of professions. Healthcare professionals were sought from a range of the types of professionals that were likely to have some involvement with a TRC (e.g., genetic counsellors, midwives, maternal fetal medicine specialists, obstetricians, etc.). Recruitment occurred until saturation was reached.

Participants were initially contacted based on the researchers’ professional networks and knowledge of their involvement in a TRC process. Potential participants were contacted by LDC and/or HBS by email or phone. Snowball sampling was then used to expand the sample. In addition, some individuals involved in TRCs made contact with the lead interviewer (HBS), expressing a desire to be interviewed for the project. If the research team were unsure about the existence of a TRC at a particular hospital, one member of the team would contact a healthcare professional at the hospital to confirm whether they have a TRC.

### Data collection

Semi-structured interviews were conducted by one or two interviewers (HBS, LK, AOR). These interviews were digitally recorded and transcribed verbatim. The majority of interviews were conducted in person, generally taking place at the healthcare professional’s office or place of practice. Some were conducted via video conferencing. Interviews were conducted from September 2019 to July 2020.

An important aspect of the data collection in this study is the involvement of ‘insiders’ in the research team [38]. The interviews were conducted by ‘outsiders’, but recruitment in the initial stages of the project was led by an ‘insider’ (LDC). Thus, this project contains elements of both ‘insider’ and ‘outsider’ research [38], with the outsiders being ‘vouched for’ by the involvement of an insider.

During the interviews, participants were asked about why they thought the TRCs existed, the decision-making processes of clinicians and the committee, the experience of the service users, related ethical issues, and the general delivery of LTOP services. In this paper, we focus on the data related to the purpose and function of the TRCs. Other results from this study are intended to be published elsewhere.

### Data analysis

Interview transcripts were analysed using inductive thematic analysis [39]. Data were co-coded by three researchers (HBS, CMH and LK) using the qualitative data analysis software, NVivo [40]. Firstly, the researchers read and familiarised themselves with the initial transcripts. They then developed a coding framework based on the themes constructed from the data. HBS and CMH then applied the coding framework on 5 transcripts to test the adequacy of the framework. Following the test coding, the coding framework was reviewed and refined through discussions between the researchers (HBS, CMH and LK), ensuring a cohesive approach to data analysis. All the transcripts were then coded by either HBS or CMH according to the refined framework. Coded data were then further analysed by HBS, CMH and LK; together they developed a collaborative coding framework to summarise the data in each code. The results presented here are the data for the code “purpose and function of the TRC.”

### Data presentation

To reduce the risk of identification in this particular study, we have opted not to include participant numbers or codes. The number of participants who are quoted is 19, with 10 participants quoted more than once; they represent a range of professions. Accompanying each quote is a broad description of the participant’s occupation. Participants are either described as ‘doctor’ or ‘other healthcare professional’; the category of ‘other healthcare professional’ includes professionals such as genetic counsellors and midwives. All participants were given the opportunity to review any quotes included in this publication.

## Results

The final number of participants was 27. The profession of the participants is presented in Table 1, and their practice in Table 2. Eighteen (67%) of the participants were women, and nine (33%) were men. The authors, wishing to ensure an accurate reflection of current practice, consulted at least one participant from all Victorian public hospitals with a TRC. There is additionally one participant from a private hospital with a TRC. Several of the participants who work at a hospital with a TRC, also work at hospitals without a TRC.

**Table 1:** Profession of participant

Type of professional	Category	*n* = 27
Obstetrician/gynaecologist/maternal–fetal medicine specialist	Doctor	15
Genetic counsellor	Other healthcare professional	6
Clinical geneticist	Doctor	2
Midwife	Other healthcare professional	2
Psychiatrist	Doctor	1
Neonatologist	Doctor	1

**Table 2:** Practice of participant

Type of practice	*n*^a^
Tertiary public hospital	19
Other public hospital	8
Private hospital	1

^a^ N.B. some participants work at multiple hospitals with TRCs, therefore n != 27

All public hospitals that carried out LTOPs had a TRC. One hospital did not do TOPs after 24 weeks; however, they were included as they had a TRC for TOPs at earlier gestations with pertinence to LTOP service delivery at other hospitals. Only one participant was primarily based at a hospital outside the Greater Melbourne area. Healthcare professionals in several other large Victorian rural public hospitals were contacted to confirm that they did not have a TRC in their respective hospitals and the scope of their TOP service delivery at the time of data collection.

We identified several key themes in the data relating to the purpose and function of the TRCs. Some participants discussed the history and justifications for the creation of the TRCs, with the Case identified as a key driver.

A further key overarching theme identified in the data was the ‘protective’ function(s) of TRCs. The functions were to protect: (1) outward appearances; (2) inward functionality; and/or (3) service users. Function (1) could mean protecting the hospital’s reputation, with the ‘Herald Sun test’—whether the TOP would be acceptable to readers of the Herald Sun, a Victorian tabloid newspaper–used as a heuristic. Function (2) related to logistics within the hospital and protecting the psychological wellbeing and personal reputation of healthcare professionals. The final function (3) related to ensuring patients received a high standard of care.

### Background

Before presenting the results on purpose and function, it is appropriate to give some more detail about the context of these committees, from the point of view of participants. There were three points that came through in many of the interviews which provided an important backdrop to the purpose and function: (1) conflicting versions of the history and/or trigger for the committees being set up; (2) lack of pathways for LTOP on psychosocial grounds in Victoria, which meant that the majority of interviews addressed only LTOP for fetal anomaly, and; (3) varied reflections on the justification for having a TRC for this procedure, given this type of process is not common in many other areas of medical practice.

### History

Some participants referred to the Case as being part of the impetus for the creation of the TRCs, which is a view supported in the previous research on this topic. However, participants had differing, and at points conflicting, views about whether the driver of the committee creation was the clinicians or the hospital management.

*“It was set up in response to [the Case] that was controversial at the time, and the hospital set up a committee that was … no one really knew exactly what it was, but we all got the feeling it was just a place so the hospital would know what was happening in the clinic. And it was, I think, partly couched as, sort of, to support the clinicians in the decision making or not, but I think over the years, that’s morphed a little bit here, there and everywhere…”* [Doctor]

*“I think, in response to concerns of the senior medical staff. So … the senior medical staff … had threatened to resign over the whole management of [staff involved in the Case]*, *but it was probably within a year or two of that. And it was in response to that. And it was a recognition that the institution had not provided the appropriate level of support.”* [Doctor]

Other participants suggested that the creation of TRCs stemmed from the 2008 Victorian law reform, although there is evidence to suggest that TRCs had at least existed at two public tertiary hospitals since approximately 2000[20]. Another participant described the TRC as ‘coming into its own’ after the reform.

*“I do think it was probably at the time of the legislation changing … I think up until then we'd sort of been working, I think, in a little bit of a grey zone …”* [Doctor]

*“… the Termination Review Panel really came into its own after the change in the law. It became even more vital at that point, because the law deliberately did not speak to gestational age, and so there is no gestational limit to a legal termination. And the lawmakers listened to what clinicians were saying and saying you need to do this on a case-by-case basis, and you need to let clinicians and institutions work it out.”* [Doctor]

Some participants described the creation of a TRC at their hospital as a natural evolution following the introduction of TOP/LTOP services at their institution, and that having a TRC was now part of ‘best practice’ for hospitals.

*“I think the fact … that it was known that we were going start having tertiary status and that we would be looking after the smallest babies, and I think with that came the expectation that we would do our own terminations … we looked through all the literature to be sure we were doing what was best practice.”* [Doctor]

*“Yeah, it’s really just the evolution of our tertiary services.”* [Doctor]

### Indication for the procedure: psychosocial reasons vs fetal anomaly

A key piece of information gained through the process of interviewing was the status difference between LTOPs based on the reason for the termination. Only one of the hospitals had a pathway for LTOP for ‘psychosocial’ or ‘social’ reasons, and this was a recent development at the time of data collection. A ‘psychosocial’ case would be any situation where the primary motivating factor for the LTOP was not a fetal anomaly. LTOP for fetal anomalies were perceived as legitimate procedures and appropriate to be performed in some hospitals; however, this was rarely the case for terminations for other reasons, despite being permitted by law.

*“Now I am being asked to present quite a number of the ones over 24 [to the TRC]*… *So [psychosocial TOP] is kind of new territory … it [the patient’s request] would just be rejected. But the government has called the hospital on it … So, it’s kind of new… the TRP after 24 weeks, it’s pretty new.”* [Doctor]

One participant, who was involved in performing LTOPs, indicated that they would not perform psychosocial LTOPs, only LTOPs where a fetal anomaly is involved. They highlighted that while the service may be lawful, ‘in reality’ there are few practitioners who are willing to perform them.

*“The other side of which we haven’t touched, we don’t know and seem to work on is social termination, you know? You’re [legally entitled] to a termination any time. I get telephone calls all the time from [around Australia] ‘would you do it for me?’ And one lady even proposed coming here from [interstate], have the injection of the baby and then go back [interstate] and tell the doctor the baby hasn’t moved and pretend nothing happened. And then they say it’s fetal death in utero without telling them what happened. I said ‘I’m not going to do that.’ So, I don’t do social termination ... I couldn’t do it. I wouldn’t be interested. I wouldn’t do it. And the legislation prohibition is, you know, you’re allowed to do it, but in reality, no one does it …”* [Doctor]

One participant implied that social grounds would not be considered ‘appropriate’ according to the law.

*“I guess termination of pregnancy, it’s offered in the situation where, I guess, according to the law, where it’s deemed appropriate, and so in general, and our principle here is that we don’t offer terminations of pregnancy purely on social grounds, on requests for termination of pregnancy because of an unwanted pregnancy. So, we don’t offer those, and we don’t even offer those in early pregnancy. It’s something that this health service just doesn’t have, a service to offer termination of pregnancy on routine social grounds.”* [Doctor]

### Justifications for the committee

Participants had differing perspectives on whether the committee’s existence for LTOP specifically could be justified, considering how rare a committee like this would be for many other medical procedures.

Some participants described the TRC as a ‘gatekeeper’ or ‘absolute barrier’.

*“… it provides that kind of space … for the decision to be made and for there to be collaboration and discussion around access … Well, it is the gatekeeper … So I think its role is that it's, yeah, a gatekeeping process, whether it should be that or not I'm not sure.”* [Doctor]

*“… the termination review panel, it sounds … almost like this sort of absolute barrier, and look, in practice, yes it could be, because you can’t do a termination within the hospital, legal though it might be, without having gone there.”* [Doctor]

Some participants described a tension regarding the terminology used to describe TRCs. Some felt it was disingenuous to refer to the committee as an ‘ethics’ committee, and others felt that the committee should provide a robust ‘ethical framework’ for decision making, but that this was not what always occurred in practice.

*“I guess I’m a bit cynical about this, and I think that the hospital’s more interested in covering its arse than providing a robust ethical framework, but I think you could also say that, yes, it’s to put a robust ethical framework in place so that we’re consistently doing the same thing and making appropriate decisions … I think its key role is an ethical decision-making body … but I don’t think that that’s how it’s being used.”* [Doctor]

*“… they would think that it should be—well, they regard it as an ethics committee some of them, they talk about it as being the termination ethics committee. It’s not an ethics committee, as far as my understanding of ethics committees go.”* [Doctor]

Others linked the TRC to legislation, but as described above, the link between the instigation of the TRCs and the decriminalisation of TOP is not straightforward. Some felt TRCs were necessary to ensure the law was being followed, suggesting that was the reason behind specifically having a committee for LTOPs and not other medical procedures.

*“So TRC is not to resolve disputed clinical issues. It’s to … make sure … the law’s being upheld.”* [Doctor]

*“And I think that the termination review panel is there to assess whether it meets the criteria of the Act, which is not about the severity, so it’s whether it meets the criteria of the Act, that it’s appropriate in all circumstances ...”* [Doctor]

*“I don’t see that the TRP role these days is to sit in judgement as such. It’s just to confirm that the case request in front of them ... is compliant with the legislation, so to make sure that everything has been done appropriately ...”* [Doctor]

### Purpose and functions

Data on participants' views relating to the purpose and function of the TRCs converged around three key functions: protecting either (1) outward appearances; (2) inward functionality; and/or (3) service users. Most participants described multiple functions for the committee, and many discussed all three as functions that the TRC plays in the hospital.

### Outward appearances

Comments that related to protecting the reputation of the hospital were classified as protecting ‘outward appearances’. For some, this meant that the TRC’s role was to prevent the types of LTOP procedures that, if they became public, would lead to adverse publicity. For others, this meant that even if the procedure itself may be viewed negatively by the public, the existence of the committee can be used to reassure the public that due consideration was given to the decision and that there were official processes in place to approve LTOPs. Multiple participants referred to ‘the Herald Sun test’, where decision-making is based on whether the particular case would be controversial enough to end up on the front page of the Herald Sun, a Victorian tabloid newspaper.

*“Because I think they’re worried about the hospital’s reputation … We call it the Herald Sun Test.”* [Doctor]

*“Ensuring that the hospital is protected from bad press, I guess. We, sometimes in our black humour of fetal medicine clinics, we talk about, is it going to pass the front page of the [Herald Sun], when we think about, if we’re going to go down this path and do a termination for this indication, and if the media get hold of it for whatever reason, does that pass that public acceptability test of having done due process, due clinical assessment …”* [Doctor]

*“Like being dragged through the mud in the headlines of the newspapers because of questionable decision-making. So, when there’s a formal committee with terms of reference and it’s carefully thought through, it’s much harder to say ‘oh, well, they did something wrong’.”* [Doctor]

*“But hospitals have put in committees, which I see is basically arse-protection and they’re for the hospital. I don't think they do anything to protect the women. And it's basically to protect the hospital against the right to lifers and various crazed politicians who will attack them with horror stories.”* [Doctor]

Other participants highlighted that TRCs may particularly benefit the hospital executive through transparency of decision-making around LTOP procedures. This is important for members of the hospital executive and leadership, who may have to account for an LTOP. A committee means there is a paper-trail and a clear decision-making process, so they are not ‘caught flat-footed’.

*“It probably meets the hospital’s governance requirements as well because I know they would be uncomfortable with just me going yay or nay. They like structure, so it does fit into that governance structure as well.”* [Doctor]

*“… like my executive do not want to be caught flat-footed with someone saying I’ve heard you’ve just done a termination at 32 weeks for a cleft lip, or whatever else.”* [Doctor]

Some participants took a slightly more positive view of the protection of outward appearance. They felt that negative publicity would not only harm the hospital and individual doctors but could also harm the chances of the service being offered to patients in the future. These participants described one of the purposes of the committee as ‘future-proofing’ the service, by protecting it from external pressure and reputational damage, to ensure it continues to be available for patients.

*“So, I think in some respects this perhaps helps to protect, to future-proof this service always being available … I guess we’ve seen examples of where suddenly a health witness has weighed in or whatever and said, ‘well, we’re not doing that anymore’, and it’s a very reactionary thing to a particular anecdote … from my experience of having been placed in the middle of it. Was it always perfect? No. Did I always love it? No. But do I think it might've been the least bad choice in terms of, as I say, just future-proofing that service for all women of the future, I think maybe it did have a useful role.””* [Doctor]

*“And if someone hasn’t seen a psychiatrist, for example, and then goes off and commits suicide or something down the track … [that] can be used as ammunition, for want of a better word, against this type of care.”* [Doctor]

### Inward functionality

Comments that related to the TRC playing a role in ensuring that the hospital and the staff within functioned as well as possible were classified as protecting “inward functionality”. A logistical purpose of the panel that some of the participants described was ensuring that staff were available to perform the procedure, and that the service was not over capacity.

*“Is someone prepared to do the feticide? There’s no point a committee sitting there in their ivory tower saying, ‘yeah, she can have a termination’, if every ultrasound technologist in the place says, ‘I’m not prepared to do a KCl [potassium chloride injection] for a missing finger’, whether we like it or not, if there’s no one prepared to do it ...”* [Doctor]

*“So, when I say capacity, I really mean: do we have staffing who could cope with the decision and managing the case? Do we have enough room? And do we have the infrastructure and the setup such that it can be looked after?”* [Doctor]

*“I think the role of the midwife on that is quite important because every now and again you have to go—we’ve got, I don't know, something else happening in birth centre on that day. Can we do it the next day or something like that …”* [Other healthcare professional]

The importance of protecting staff wellbeing was also stressed by multiple participants, ensuring that the performance of an LTOP did not have an excessive psychological impact on staff, particularly the midwives who assist in delivery.

*“… the midwives are the ones who’re right at the coalface in that regard, and when it’s a late termination, because they’re delivering a baby that is going to either die there in the labour ward or has already been given a lethal injection and is already going to be dead, so they’re delivering a dead baby. So, psychological impact.”* [Doctor]

*“So, terminations are usually done on birth suite, so you can have a woman terminating her 26-week baby in one room and in the next room is a woman desperately trying to not deliver her 23-week baby. And I understand that midwives have trouble reconciling those two things. The same midwife might be asked to look after those two rooms, it’s one to two, and that is a really hard ask. In one room you’re agreeing to not have a baby, and in another room you’ve got someone saying, ‘I’ll do anything to save this baby,’ and it does cause staff a lot of distress.”* [Doctor]

*“It’s also the challenge around, the person doing the feticide can’t be seen just as the proceduralist, otherwise they then really do see themselves as the executioner and so they’ve got to have some involvement in the level of comfort that they’ve got for things like that too.”* [Doctor]

Some participants viewed the purpose of the committee as existing to support the individual clinicians and other staff from taking sole responsibility for the decision, so that they do not feel like ‘judge, jury and executioner’. It was also suggested that the panel ensures that the hospital supports the decision being made and provides protection for individual clinicians. It is also implicit in some of the comments that the protection offered by the committee process is connected to the outcome of the Case and the impact on the clinicians involved at the time.

*“And I think it gives us great comfort to know that a group of senior clinicians in the hospital, independent of us, have heard everything, and they too agree that this is an appropriate course of action, and that the hospital supports it, because that way if something happens … at least we know that we have gone through this formal process where others have heard the full story, and have agreed that this is something that the institution can support. So, I think having that institutional backing is important … otherwise you do sometimes feel a bit like, sort of, literally judge, jury and executioner, where you’ve made the diagnosis on the scan, perhaps, you’ve counselled the patient, and then you’re the one doing … It’s like, it might be nice to have someone else just to agree that this is all okay.”* [Doctor]

*“… you know, it’s a decision by a committee, so it’s not an individual doctor that ends up having to have their reputation tainted by the media or that kind of thing. So, [I] think it’s a bit of a protection for the doctors involved.”* [Other healthcare professional]

*“I never ever want to see someone hung out to dry like Lach was [in the Case]. That’s what it’s about. That’s what the committee’s all about. So, nobody gets hung out to dry like Lach was and his colleagues.”* [Doctor]

*“I think that it makes all the staff who are dealing with the women feel as though it's been through a higher process, if you like, and so they feel a bit more comfortable because they know that there’s been robust discussion about it and that they’re not going to get their name in the paper, that sort of thing.”* [Other healthcare professional]

### For users of the service

The final purpose and function of the committee reported by some participants was for the users of the service, rather than for either the hospital or the staff working in the hospital. While some have commented in previous quotes that the committee is not there for patients (e.g. “*I don't think they do anything to protect the women”* [above]), others argued that one of the functions of the TRC was to protect users of the service. Some participants described this as a means to ‘tick all the boxes’–ensuring that all opinions and options have been considered, the patient has been appropriately counselled, and that all the appropriate tests and steps have been taken. This helps ensure that due process has been followed and the patient has received the appropriate care. This gives the TRC a ‘protective’ role for the users of the service.

*“So, I think it’s actually a valid process for that reason, as well, it actually is protective and it is just another check and balance to make sure that everyone has ticked all the boxes because if you don’t have this oversight in complex systems things get missed …”* [Doctor]

*“But I feel that they are more there to make sure that we have done the right thing, that we have given the woman all the information that she needs to have. And that, basically, all the requirements have been met for her to come to the decision that she wants to make… I think the committee exists to protect the couples, so to make sure that they get the right information …”* [Doctor]

*“So, I see a key role of the committee, maybe it’s an opportunity to consolidate thoughts about things, to explore all the avenues. It doesn’t hurt to have gone through the checklist to make sure that you’ve done everything, and explained it all, and that is probably not a bad thing.”* [Doctor]

Some participants went even further to suggest that in fact a primary function of the TRC is to provide the best possible care for users of the service, rather than the protection of reputation or ensuring smooth hospital functioning.

*“… the governance is overseeing the clinical practice to make sure that the family are getting the best possible care, yeah, I probably would set up something like that.” [Doctor]*

*“… now really everybody's onboard [with] this idea of this is good practice and this is good care of our patients, who are requesting late term abortions ... we do it for them, not to protect the hospital.” [Other healthcare professional]*

*“Compared to when I started, it was so fragmented for the women. It was just horrible. They would turn up in the middle of the night, no one would know about them. The conscientious objections thing, there was just no coordination. So, I think we’ve fixed that, so I’m happy with it.” [Doctor]*

## Discussion

Regulation of TOP provision is undergoing significant reform across many jurisdictions globally, impacting the accessibility of the procedure. Beyond law, a range of barriers—such as financial cost to patients—also impact accessibility. Institutional processes and policies can impact TOP provision and accessibility more directly in relation to service delivery. This study, and the data presented in this paper, explored healthcare professionals’ views on the purpose and function of TRCs (a form of institutional process) in Victoria, Australia. Overall, a key theme that emerged from the data was the ‘protective’ function of TRCs. Many participants stressed the (i) protection of the hospital reputation and (ii) protection of the healthcare professionals involved in the process. Others also highlighted the protective function for (iii) service users. These findings have implications for other regions and jurisdictions globally in relation to managing TOP and LTOP provision at the institutional level. Indeed, as this study has demonstrated, care pathways for LTOP are not only influenced by law (and broader clinical guidelines), but are also significantly affected by institutional processes and decisions made therein.

A critical insight that has emerged from our data is the role that external actors and societal pressure play in institutional decision-making. This has arguably resulted in the TRC playing a role in safeguarding the hospital reputation, as well as the personal reputation and wellbeing of healthcare professionals. Multiple participants referred to the risk of an LTOP case coming to the attention of the media, specifically the Herald Sun. The Herald Sun, a Victorian tabloid newspaper, has been described as having a broadly conservative influence on public debate [41], and has reinforced anti-TOP narratives in the past [42]. A columnist for the Herald Sun, Andrew Bolt, had repeatedly discussed the Case and criticised the actions of the doctors [43]. Since that time, scrutiny of policy and healthcare provision has increasingly been found to occur through social media platforms such as Twitter, not just traditional media outlets such as newspapers [44]. There has been an increasing focus on developing policies and strategies for healthcare systems in relation to social media [45]. One study examining the social media policies of National Health Service (NHS) Trusts in England found that they focused on protection of institutional reputation, which was at odds with the national strategy [46]. Hospitals, in the same way as other public sector organisations, increasingly need to engage in reputation management to address the potential impact of negative perceptions on services [47].

This view held by some participants that the TRC was a means of protecting against reputational damage from the media may have been influenced by the extensive, and often negative, media coverage of the Case that also specifically targeted some clinicians [48, 49]. As outlined by participants, the TRC can play a role in reputation protection here in two ways: it can prevent contentious LTOPs that may affect a hospital’s reputation from being performed; or, it can provide a defence that there had been “due process” and that decisions relating to LTOP had been “carefully thought through”, which may help to mitigate any reputational damage that may arise in such cases in the event they receive media attention. In this way, policy can serve as a “rhetorical strategy” to legitimise a certain course of action as well as framing discourse in a specific way that reinforces the power and role of the institution [50].

It is important to recognise that, according to some participants, the Case was the impetus for the creation of some TRCs—such as for the Royal Women’s Hospital and Monash Medical Centre, as previously described [20]—but for more recent TRCs, the impetus for the creation may have been different. For example, some participants described the creation of a TRC as coinciding with LTOP services being offered at a particular hospital, and that it was “best practice”. Some of the participants indicated that they had been in clinical practice at the time of the Case and were intimately familiar with what happened, while other participants did not have knowledge of the Case. Therefore, participants’ differing views on the reasons for the creation of the TRC may be partly influenced by whether they were in clinical practice at the time of the Case, whether they were involved in the Case or witnessed the impact on the medical practitioners and the hospital involved, and/or how recently their hospital began offering LTOP services. This may represent a form of loss of “institutional knowledge”, where information is not passed down to newer members of an organisation or institution [51]. However, even where the impetus for the creation of a TRC was related to the Case, it is important to recognise that the purpose and function of any particular TRC may have evolved over time. Organisational change within institutions can be shaped by changing societal values and norms, which is particularly relevant in the context of changing societal views and discourse surrounding TOP [52].

Using ‘The Herald Sun test’ to decide whether to provide medical care may negatively impact patients who are seeking an LTOP. In particular, this may have been the reason that at the time of data collection only one hospital was offering LTOPs on the basis of “purely” psychosocial grounds, which was described as occurring only after the application of government pressure. The majority of data collected in this study related to LTOPs for fetal anomalies. The lack of provision for psychosocial termination may be traced to the reputation-preserving function that the TRCs hold. This is because LTOPs for psychosocial reasons may be seen as less “deserving”, with some participants implying that it was not an “appropriate” reason for LTOP under the law, even though there is no legal basis for this opinion. There appeared to be a continuum of “acceptability” or “appropriateness”, where maternal or psychosocial reasons are seen as less or not acceptable; minor fetal anomalies are on the borderline; and ‘severe’ fetal anomalies are acceptable. We will explore this more fully in a separate paper.

Overall, this reputation-preserving function of the TRCs—‘the Herald Sun test’—is a key concern when addressing the provision of LTOP services. Although the decision-making processes of the TRCs have not been described extensively here, their suggested function of preventing reputational damage indicates a possibly inappropriate means of managing decision-making surrounding LTOP at the institutional level. These results suggest that media criticism can influence institutions’ management of controversial medical procedures such as LTOP, rather than relying on evidence-based practice or other ethical considerations, such as what is best for the patient. This allows influential media figures to have an outsized impact on institutional approaches to these cases, where the patients are likely to be highly vulnerable and distressed. This is a serious concern in this context.

A comparison could be drawn between the Case and other controversial cases or decisions in health care. For example, in the past decade there have been several high-profile disagreements relating to life-sustaining treatment for critically ill children in the NHS, such as those of Charlie Gard and Alfie Evans [53]. These cases impacted public trust in hospitals, and also resulted in healthcare professionals being subject to criticism in addition to receiving death threats. For these reasons—particularly damage to public trust—there may be an ethical justification for organisational responses that are aimed at mitigating reputational damage, even if they may not otherwise be the most appropriate or standard responses for a particular case [54]. The importance of public trust in healthcare systems has also been highlighted by the COVID-19 pandemic and the impact of public trust on effectiveness of policy responses [55]. This perspective can align with some of the views expressed by participants in this study, who described how protecting the hospital’s reputation and mitigating controversy can actually “future-proof” LTOP service delivery. However, it is important to recognise that cases such as those of Gard and Evans involved public pressure on clinicians to continue to provide certain treatments, with the view that this is in the patients’ best interests, and the parents as surrogate decision-makers [56]. In the context of TRCs and LTOP service delivery, the desire to mitigate controversy may lead to autonomous individuals being refused a medical intervention, a decision which is not in their best interests, to secure public trust and/or protect the reputation of institutions.

In addition to the reputation of the hospital, participants also viewed the TRC as protecting the personal reputation and wellbeing of clinicians. Because the TRC has multiple members, it removes the individual responsibility from specific clinician(s) and instead situates the responsibility for the decision at a group or institutional level. This can serve to protect clinicians in multiple ways. As one participant outlined, making decisions around LTOP as a committee protects individual clinicians’ reputations, including if an LTOP case comes to the attention of the media. Qualitative research with fetal medicine specialists in the Republic of Ireland illustrated a similar sense of caution, with one participant stating *“…it was fetal medicine reports on the front page of the newspaper being read out in the Dáil [Irish Parliament], that is at the back of your mind” *[57].

The TRCs can also serve as a form of psychological protection from responsibility for the decision-making, which makes clinicians feel more “comfortable”; this prevents clinicians from feeling like, as one participant described, “judge, jury and executioner”. It is interesting to note that this language positions the clinician as the ‘executioner’ of the fetus, although it could also potentially refer to being the ‘executioner’ of the procedure in general. Thus, displacing responsibility for decision-making from clinicians to an institutional process can potentially prevent moral distress or discomfort that clinicians may otherwise feel when involved in an LTOP case. The occurrence of moral distress in nursing practice for TOP provision has been previously described in the literature [58, 59].

The wellbeing of midwives, as workers “at the coalface”, was also frequently highlighted by participants. This is because participants indicated that midwives and/or nurses find delivery after a feticide distressing, particularly as it often occurs on the same birth ward as live births. In this way, midwives are “working with birth and death in the same space” [60]. This can be the case even where midwives support the decision that the patient or couple has made regarding LTOP [61]. In addition to their own potential distress, midwives may also need to navigate the grief and emotional responses of the patient or couple. Qualitative research from Denmark and Sweden has reported how midwives perform “rituals” such as wrapping the aborted foetus in cloth, offer the chance to say “goodbye”, and recognise the couple as parents [61, 62]. Provision of LTOP-related care by midwives may be associated with occupational stigma and increased likelihood of burnout [63]. Here, the TRCs serve the function of mitigating the impact on midwives’ wellbeing by ensuring that the timing of the LTOP was appropriate and that there was staffing capacity, which may in part be influenced by the willingness of staff to be involved.

Some participants expressed scepticism as to whether the stated or purported roles of the committees (for example, in its Terms of Reference) were in fact its real purposes. For example, one participant described the hospital as being more interested in “covering its arse” than providing a “robust ethical framework”. Participants also highlighted tensions between its perception and possible role as an ‘ethics committee’, and the way the TRCs function in practice. However, even if the TRCs were to be understood as a type of ethics committee, it is important to recognise the way that such committees can nonetheless also function to protect the reputations and interests of institutions such as hospitals [64, 65].

A key question is how the implementation of the TRCs differentiates LTOPs from other kinds of medical procedures. Some participants referred to TRCs using phrases such as “gatekeeper” or “barrier”. In this way, the institutional requirement for TRC approval can function as a barrier to LTOP access. Qualitative research with healthcare professionals in other contexts have highlighted that provision of TOP services is viewed to be part of normal and routine healthcare [66]. However, the use of the TRC may position LTOP as a service that is non-routine and requires special approval. These findings can be used to inform policy and practice relating to TOP and LTOP services for a variety of actors in a range of contexts. These data provide a deeper understanding of some of the ways in which institutional provision of services can be impacted or shaped beyond law. While non-legislative barriers to TOP access are well documented in the literature, this study provides a closer examination of the role of formalised institutional processes and policies. Recognising the function and purpose of processes such as TRCs allows us to identify aspects of LTOP care pathways that could likely be improved or changed, both for patients and healthcare professionals. Participants’ perceptions in this context can inform the broader development of policies that appropriately avoid the potential negative impact of such institutional processes (such as being a “gatekeeper” for patients to access care), but also integrate the supportive or positive functions as identified by participants (such as facilitating staff wellbeing). However, to do so may potentially involve trade-offs between the interests of different parties.

Another salient finding from this study is how perspectives and knowledge about the TRCs varied amongst participants, across professions, career stages, and institutions. There was also heterogeneity amongst participants about interpretations of the legislation, and when an LTOP might be considered “appropriate”. This may support the concern in various healthcare systems internationally about how the “postcode lottery” can affect access to TOP services, as well as healthcare generally [67, 68]. Thus, even in a jurisdiction where legislation allows for TOP at all gestations, there can be the development of formalised—but not necessarily consistent, standardised or justified—institutional processes, which can impact care.

There are a number of limitations that impact the conclusions drawn from this study. The purposive sampling approach may have led to bias in views from participants with particular perspectives on TRCs, particularly as some participants requested to be interviewed as opposed to being invited. Some participants’ responses may also have been affected by a personal relationship with one of the researchers, which is a recognised challenge with insider research; for this reason, that researcher did not participate in interviews or data analysis [69]. Furthermore, with only one participant from a regional area, the data generally only speak to the delivery of LTOP services within the Greater Melbourne area in Victoria. While regional patients requesting LTOP are generally sent to Melbourne, participants’ views may not be applicable to general delivery of TOP services across the state. This may also impact the degree to which these findings have implications or can inform practice in other jurisdictions and healthcare systems.

## Conclusion

The results of this study have implications for how we should understand the delivery of LTOP services in Victoria, Australia, as well as policy and processes in other jurisdictions. They provide further clarity on the processes involved in LTOP provision from the perspective of the healthcare professionals involved. This is a case study of how TRCs are used in LTOP delivery within a regulatory context where there is no gestational limit on TOP, but nonetheless institutional processes are developed which may function as “barriers” for LTOP access. Within a social context where LTOP remains stigmatised and contentious, TRCs appear to primarily protect institutions and clinicians, with some benefits and disadvantages for service users. A key challenge for service delivery in the future is to reckon with how societal pressure—including media coverage—should influence both institutional and clinical decision-making around procedures such as LTOP. These findings can inform policy and practice in other jurisdictions and healthcare systems.

## Data Availability

The data generated and/or analysed during the current study are not publicly available in order to maintain the confidentiality of participants’ identity.

## Abbreviations

TOP: Termination of pregnancy
LTOP: Late termination of pregnancy
TRC: Termination review committee
TRP: Termination review panel
HREC: Human Research Ethics Committee
